# Poor outcome of pediatric B-cell acute lymphoblastic leukemia associated with high level of *CRLF2* gene expression in distinct molecular subtypes

**DOI:** 10.3389/fonc.2023.1256054

**Published:** 2023-11-07

**Authors:** Danna Lin, Keyan Yang, Lihua Yu, Lulu Huang, Xiaorong Lai, Li Wu, Xiayu Xia, Jingwen Zhang, Qinlong Zheng, Lihua Yang

**Affiliations:** ^1^ Department of Pediatric Hematology, Zhujiang Hospital, Southern Medical University, Guangzhou, Guangdong, China; ^2^ Laboratory of Molecular Diagnostics, Beijing GoBroad Boren Hospital, Beijing, China; ^3^ Department of Clinical Hematology&Flow Cytometry, Guangzhou KingMed Center for Clinical Lab. Co., Ltd., Guangzhou, China

**Keywords:** B-ALL, CRLF2, Ph-like, pediatric, leukemia

## Abstract

**Background:**

Overexpression of the cytokine receptor-like factor 2 (CRLF2) gene is the most common feature in the Philadelphia chromosome (Ph)-like subtype of B-cell acute lymphoblastic leukemia (B-ALL). However, the predictive value of *CRLF2* overexpression for the prognosis of pediatric B-ALL patients remain controversial. The molecular mechanisms that upregulate *CRLF2* expression level in patients has not been fully elucidated.

**Methods:**

In this study, the prognostic impact of *CRLF2* expression level on molecular types of B-ALL in pediatric patients from Zhujiang Hospital (n = 111) was retrospectively analyzed. Youden index analysis was used to categorize *CRLF2* expression into 3 groups, and these categories more precisely described the differences in the prognosis of patients with varying expression levels of *CRLF2* in both the Zhujiang Hospital cohort and the TARGET cohort.

**Results:**

We used the Zhujiang Hospital cohort as a discovery cohort to determine the cutoff value of *CRLF2* expression. *CRLF2*-high patients accounted for approximately 6%. In addition, the percentage of bone marrow blast cells and initial white blood cell count in *CRLF2*-high patients were higher than those in *CRLF2*-low patients, and MRD turned negative slower. The results were validated in the TARGET cohort and indicated that *CRLF2* overexpression could be subdivided by *CRLF2* expression levels into 2 categories: *CRLF2*-high with a poor survival and *CRLF2*-medium with a good OS and EFS. Such heterogeneity was attributed to the different molecular mechanisms leading to *CLRF2* upregulation, where the *CRLF2* overexpression level was high in Ph-like B-ALL and medium in high hyperdiploid B-ALL.

**Conclusion:**

This study highlights the importance of the molecular mechanisms of the upregulation of *CRLF2* expression in predicting the prognosis of pediatric B-ALL patients.

## Introduction

The cytokine receptor-like factor 2 (*CRLF2*) gene, located on chromosome Xp22.3 or Yp11.3, encodes cytokine receptor-like factors (CRLFs), which form heterodimers with IL-7Ra that receive and transmit signals from thymic stromal lymphopoietin and upregulated *CRLF2* expression activates the Janus kinase/signal transducer and activator of transcription (JAK/STAT) or phosphatidylinositol 3-kinase/mammalian target of rapamycin (PI3K/mTOR) pathway to promote the occurrence and progression of leukemia (1–4). *CRLF2* overexpression has been reported as a major feature in the Philadelphia chromosome (Ph)-like subtype of B-cell acute lymphoblastic leukemia(B-ALL), and approximately 50-60% of patients with Ph-like B-ALL have *CRLF2* overexpression (1–4). Nonetheless, whether *CRLF2* overexpression can be employed to predict the prognosis of B-ALL patients is controversial. The majority of previous studies have demonstrated that *CRLF2* overexpression is associated with the poor prognosis of B-ALL patients (1–4). However, some studies have revealed that *CRLF2* overexpression is not an independent prognostic factor for B-ALL (4, 5). The definition of *CRLF2* overexpression varies in these studies, and the proportion of patients with *CRLF2* overexpression among unscreened patients ranges from 4.7 to 25% (2, 4–7). Different criteria for *CRLF2* overexpression may cover a variety of molecular mechanisms leading to the upregulation of *CRLF2* expression; therefore, it is difficult to precisely characterize the factors driving disease progression (7–10).

In this study of Chinese pediatric patients, we identified varying levels of *CRLF2* expression among different molecular subtypes. New cutoff value for *CRLF2* overexpression associated with molecular types were proposed. Moreover, the prognoses of patients with different *CRLF2* expression levels were validated using the B-ALL patient cohort from the Therapeutically Applicable Research to Generate Effective Treatments (TARGET) public database. Finally, the corresponding cutoffs for *CRLF2*-high in real-time quantitative polymerase chain reaction (RQ-PCR) were also determined, thereby providing a basis for the rapid identification of B-ALL patients with a potentially poor prognosis via *CRLF2* expression level.

## Materials and methods

### Zhujiang Hospital cohort and sample collection

One hundred and eleven patients with B-ALL treated at the Department of Pediatrics, Southern Medical University, Zhujiang Hospital, between May 2020 and February 2022 were enrolled in this study; the final follow-up date was August 25, 2023. Children diagnosed before 1 year of age (n=5) were treated with the Inerfant-06 protocol (11). Among children diagnosed after 1 year of age, 1 was initially treated intermittently at another hospital, 1 was treated using the Guangdong Children’s Leukemia Group-ALL-2008 (GD-ALL-2008) protocol (12), and the others were treated using the South China Children’s Leukemia Group-ALL-2016 (SCCLG-ALL-2016) protocol (12) (n=104). The detailed clinical characteristics are shown in **Table 1**. Bone marrow (BM) samples were collected from patients at initial diagnosis. This study was approved by the central ethics committee of Zhujiang Hospital, Southern Medical University, in accordance with the Declaration of Helsinki (Approval number: 2022-KY-195-01).

**Table 1:** Baseline characteristics of the Zhujiang Hospital cohort.

Characteristics		Total (n = 111)	
Sex (n, %)	Female	44	40%
	Male	67	60%
Age (n, %)	≤6	81	73%
	>6	30	27%
Karyotype (n, %)	Abnormal	35	32%
	Normal	61	55%
	Unknown	15	14%
Blasts in BM (%) (n=109)	median (IQR)	86	(76-93.25)
Initial WBC (×10^9^/L)	median (IQR)	13.44	(5.23-43.5)
RNA-seq Subtype (n, %)	DUX4	4	4%
	ETV6-RUNX1	12	10%
	ETV6-RUNX1-like	2	2%
	High hyperdiploid	38	33%
	KMT2A Group	9	8%
	Low hypodiploid	1	1%
	MEF2D	4	4%
	NUTM1	2	2%
	PAX5 P80R	1	1%
	PAX5alt	5	5%
	Ph	4	4%
	Ph-like	5	5%
	TCF3-PBX1	11	10%
	ZNF384 Group	1	1%
	Unclassified	13	12%
Risk (n, %)	SR	13	12%
	IR	36	32%
	HR	61	55%
	Unknown	1	1%
Treatment (n, %)	Interfant 2006	5	5%
	SCCLG ALL 2008	1	1%
	SCCLG ALL 2016	104	94%
	Others	1	1%
*CRLF2* FPKM group (n, %)	*CRLF2-*high	6	5.4%
	*CRLF2-*medium	47	42.3%
	*CRLF2-*low	58	52.3%
*IKZF1* ^plus^ (n, %)	*IKZF1* ^plus^	7	6%
	*IKZF1* deletion	7	6%
	No *IKZF1* deletion	97	87%

BM, bone morrow; IQR, interquartile range; WBC, white blood cell; SR, standard risk; IR, intermediate risk; HR, high risk; SCCLG, South China Children’s Leukemia Group.

### The TARGET cohort public data collection and preprocessing

Data for the TARGET cohort were initially generated for the Therapeutically Applicable Research to Generate Effective Treatments (https://ocg.cancer.gov/programs/target) initiative (TARGET Phase II, phs000464). The data used for this analysis are available at (https://portal.gdc.cancer.gov/projects). Clinical and survival data were downloaded from cBioportal (http://www.cbioportal.org).

The TARGET cohort included 144 patients diagnosed with B-cell lymphoblastic leukemia and with diagnostic tumor RNA samples. FPKM values were calculated using the gene expression counts matrix from the RNA sequencing. The clinical characteristics are provided in **Supplementary Table 2**.

### DNA target sequencing and mutation analysis

Genomic DNA was obtained from BM at diagnosis and subjected to hybridization-based capture by using a KAPA HyperPlus Kits (Roche Diagnostics, Basel, Switzerland) and then captured using a NimbleGen SeqCap EZ Library SR (Roche Diagnostics) kit probing the exon regions of 339 genes relevant to hematologic malignancies, followed by high throughput sequencing on an Illumina NextSeq 550 platform to produce PE 150 bp. Somatic variant calling was performed with the Broad Institute’s Genome Analysis Toolkit (GATK) v4.0.4.0 and Vardict v1.12 and annotated by Annovar v2018-04-16 (13–18). Selected pathogenic variants were manually inspected using the Integrative Genomics Viewer (IGV) software v2.8.0 (19).

### mRNA sequencing and B-ALL subtype classification

Total RNA was extracted from the BM samples using a Cowin RNApure Blood kit (Cowin Bio., Jiangsu, China) and quantified using a Nanodrop one (Thermo Fisher). Stranded mRNA sequencing libraries were constructed using a VAHTS Stranded mRNA-seq Library Prep Kit for Illumina (Vazyme, Nanjing, China). The polyadenylated mRNAs were then purified and fragmented, followed by the generation of double-strand cDNA. The double stranded cDNAs were ligated to the VAHTS RNA Adapters (N803/N804, Vazyme, Nanjing, China) following end repair and A tailing. The purified ligation products (size 200-500 bps) were selected and quantified, followed by sequencing on an Illumina Novaseq 6000 to produce ≥30M 150 base pair paired-end reads. After performing quality control on the sample’s raw FASTQ data, reads were trimmed using fastp v0.19.4 to remove sequencing adaptors and low-quality bases or reads (13). The preserved clean reads were then mapped to the hg19 human reference genome using Hisat2 v2.2.1 and STAR v2.7.8a (20, 21). Gene fusions were detected using Arriba v2.3.0 and CICERO v1.7.0, with suspected pathogenic fusions confirmed by PCR and Sanger sequencing (22, 23). The read numbers for each gene were counted using HTSeq tool v0.13.5 (24). The gene expression counts for Zhujiang Hospital cohort samples and the TARGET cohort samples were input into ALLSorts (28 Jul 2021) (25). ALLSorts B-ALL subtype classifier predicted 18 subtypes and 5 meta-subtypes.

### Multiplex ligation-dependent probe amplification assay

The MLPA assay was performed for all samples using 2 kits: Salsa MLPA Probemix P335 ALL-IKZF1 (MRC-Holland, Amsterdam, The Netherlands) to characterize the copy number alterations of the *IKZF1, PAX5, ETV6, RB1, BTG1, EBF1, CDKN2A, CDKN2B* genes and *PAR1* region (*SHOX* area, *CRLF2, CSF2RA, IL3RA* and *P2RY8* genes) and Salsa MLPA Probemix P327 iAMP21-ERG (MRC-Holland) to identify the copy number alterations of the *RUNX1* and *ERG* genes.

Based on the MLPA results, patients were classified into 3 groups: No *IKZF1* deletion, *IKZF1* deletion (*IKZF1*del only) and *IKZF1* ^plus^. *IKZF1* ^plus^ was defined as *IKZF1*del plus *PAX5, CDKN2A, CDKN2B* and/or *CRLF2* with *ERG* not deleted (26).

### Detection of *CRLF2* expression using real-time quantitative PCR

RQ-PCR analysis of *CRLF2* expression was performed following the reverse transcription of RNA samples using a RevertAid First Strand cDNA Synthesis Kit (K1622) (Thermo Fisher). RQ-PCR was performed using an ABI 7500 Real-Time PCR System (Thermo Fisher). The housekeeping gene *GUS* was used as an internal control. The primers and probes used for RQ-PCR analysis are listed in **Table 2**. PCR was performed in 20 μL reactions consisting of 1× TaqMan™ Fast Advanced Master Mix (Thermo Fisher), 300 nM primer, 200 nM probe and 5 μg of complementary DNA. The thermal cycling conditions were 50°C for 2 min and 95°C for 2 min, followed by 45 cycles of 95°C for 3 s and 60°C for 30 s. The ratio of *CRLF2* was calculates using *CLRF2* copies normalized to *GUS* copies.

**Table 2:** List of RQ-PCR primers and probes.

Gene	Type	5’-3’
*GUS*	F. Primer	GGAATTTTGCCGATTTCATGA
*GUS*	R. Primer	CCGAGTGAAGATCCCCTTTTT
*GUS*	Probe	AACAGTCACCGACGAGAG
*CRLF2*	F. Primer	GCAAGTCGCTGGATGGTTTATT
*CRLF2*	R. Primer	CCACGAAAATCTCACGTGCTT
*CRLF2*	Probe	CCTGAAACCCAGTTCC

### Multi-parameter flow cytometry analysis

MFC for assessing MRD was performed on whole BM specimens obtained at the specified time intervals using a standard stain-lyse-wash procedure. A total of 1×10^6^ cells were stained per analysis tube, acquired on a Navios flow cytometer using Navios software (Beckman Coulter) and analyzed using Kaluza (Beckman Coulter) or FCS Express (*De Novo* Software).

A 2-tube 7-colour panel was run. Tube A contained CD58-FITC (cLone: AICD58, Beckman Coulter), CD34-PE (cLone: 581, Beckman Coulter), CD10-PE-Cy7 (cLone: HI10a, BD), CD19-APC (cLone: SJ25C1, BD), CD38-eFluor 450 (cLone: HB7, eBioscience), CD45-BV510 (cLone: H130, BD) and 7AAD Viability Dye (Beckman Coulter). Tube B contained CD66c-FITC (cLone: B6.2/CD66, BD), CD13-PE (cLone: L138, BD), CD33-PE (cLone: D3HL60.251, Beckman Coulter), CD34-PerCP-Cy5.5 (cLone: 581, Biolegend), CD10-PE-Cy7 (cLone: HI10a, BD), CD19-APC (cLone: J25C1, BD), and CD45-BV510 (cLone: H130, BD).

MRD was identified using both the leukemia-associated immunophenotype (LAIP) and different-from-normal (DFN) method. To distinguish residual tumor B cells from normal B-progenitor cells, we used B-lymphocyte lineage and stage differentiation associated antigens such as CDl9, CDl0, CD34 and CD45, as backbone antibodies. Tumor B cells exhibit strong expression of CDl0, CDl9 and CD34, low expression of CD45, cross-line expression of myeloid antigens such as CD66c, CDl3 and CD33, overexpression of CD58 and low expression of CD38.

### Efficacy assessment

The short-term efficacy assessment indicators included the results for the response to prednisone and the results for the 15^th^ and 33^rd^ days of BM flow cytometry minimal residual disease (FCM-MRD). Response to prednisone is an independent prognostic indicator of acute lymphocyte leukemia. Prednisone good response (PGR) is considered the absolute blast cell count of peripheral blood on the 8th day ≤ 1000/μL (27). This study uses the scrag-al-2016 risk-layered system and the results of the response to prednisone, with the 15^th^- and 33^rd^-day BM FCM-MRD less than 1% and the 33^rd^-day FCM-MRD less than 0.01% (28). Complete remission (CR, Blasts cells in bone marrow <5% and no measurable extramedullary leukemia) was defined according to the U.S. National Comprehensive Cancer Network (NCCN) guidelines on day 33 (29).

### Statistical analysis

Statistical analyses were conducted using R version 3.6.2 and IBM SPSS statistics 25. Data are presented as the median and interquartile range (IQR). The Wilcoxon rank-sum test and Kruskal-Wallis test were applied to assess differences between 2 groups and more than 2 groups. Receiver operating characteristic curves (ROCs) and the Youden index were used to identify optimal cutoff values for gene expression (30). Fisher’s exact test was used to analyze the clinical baseline of patients with different *CRLF2* expression levels. Multivariate Cox regression, Kaplan-Meier curves and the log-rank test were used to analyze OS and EFS via the ‘survival’ and ‘survminer’ packages in R. *P*<0.05 was considered significant for two-sided tests.

### Data availability

The data from Zhujiang Hospital cohort used in this study can be found in **Supplementary Table 1**. The datasets an analyzed in the current study are publicly available from the ALL dataset of the TARGET database (TARGET Phase II, phs000464) [https://portal.gdc.cancer.gov/projects], and the clinical data were downloaded from cBioPortal [https://www.cbioportal.org/study/clinicalData?id=all_phase2_target_2018_pub]. The data for the Zhujiang Hospital cohort are available upon reasonable request.

## Results

### Basic information on study cohorts

A total of 111 newly diagnosed pediatric B-ALL patients admitted to and treated in the Department of Pediatric Hematology, Zhujiang Hospital, Southern Medical University, China, from May 2020 to February 2022 were enrolled in the Zhujiang Hospital cohort. The last follow-up was conducted on August 25, 2023, with a median follow-up time of 28.6 months (95%CI: 27.6-32.1 months). There were 67 (60%) males and 44 (40%) females, with a median age of 4 years (range: 0-14 years), including 81 patients (73%) aged ≤6 years and 30 patients (27%) aged >6 years. On the basis of the South China Children’s Leukemia Group-ALL-2016 (SCCLG-ALL-2016) risk stratification (28), 13 patients (12%) were standard risk, 36 (32%) were intermediate risk, 61 (55%) were high risk, and 1 (1%) had an uncompleted risk assessment due to the abandonment of treatment. At the initial visit, for all 111 patients, the median white blood cell count was 13.33×10^9^/L [interquartile range (IQR): 5.23-43.5×10^9^/L], and 24 patients had a white blood cell count ≥50×10^9^/L. Moreover, the median proportion of bone marrow blast cells was 86% (IQR: 76-93.25%) at the initial visit among the 109 patients with records (**Table 1**). Next, molecular typing of B-ALL was implemented using ALLSorts, with the following results: DUX (4, 4%), ETV6-RUNX1 (12, 10%), ETV6-RUNX1-like (2, 2%), high hyperdiploid (38, 33%), KMT2A group (9, 8%), low hypodiploid (1, 1%), MEF2D (4, 4%), NUTM1 (2, 2%), PAX5 P80R (1, 1%), PAX alt (5, 5%), Philadelphia chromosome positive (Ph+, 4, 4%), Ph-like (5, 5%), TCF3-PBX1 (11, 10%), ZNF384 (1, 1%), and unclassified (13, 12%). Six patients had the Ph-like subtype, carrying somatic mutations of *KRAS* (n=1), *FLT3* (n=1), *PAX5* (n=1), and *PHF6* (n=1) (**Supplementary Figure 1A**) as well as *CRLF2*-associated fusion (n=2) and *EBF1*-*PDGFRB* fusion (n=1).

### *CRLF2* overexpression was associated with the Ph-like and high hyperdiploid genetic subtypes

**Figure 1:**
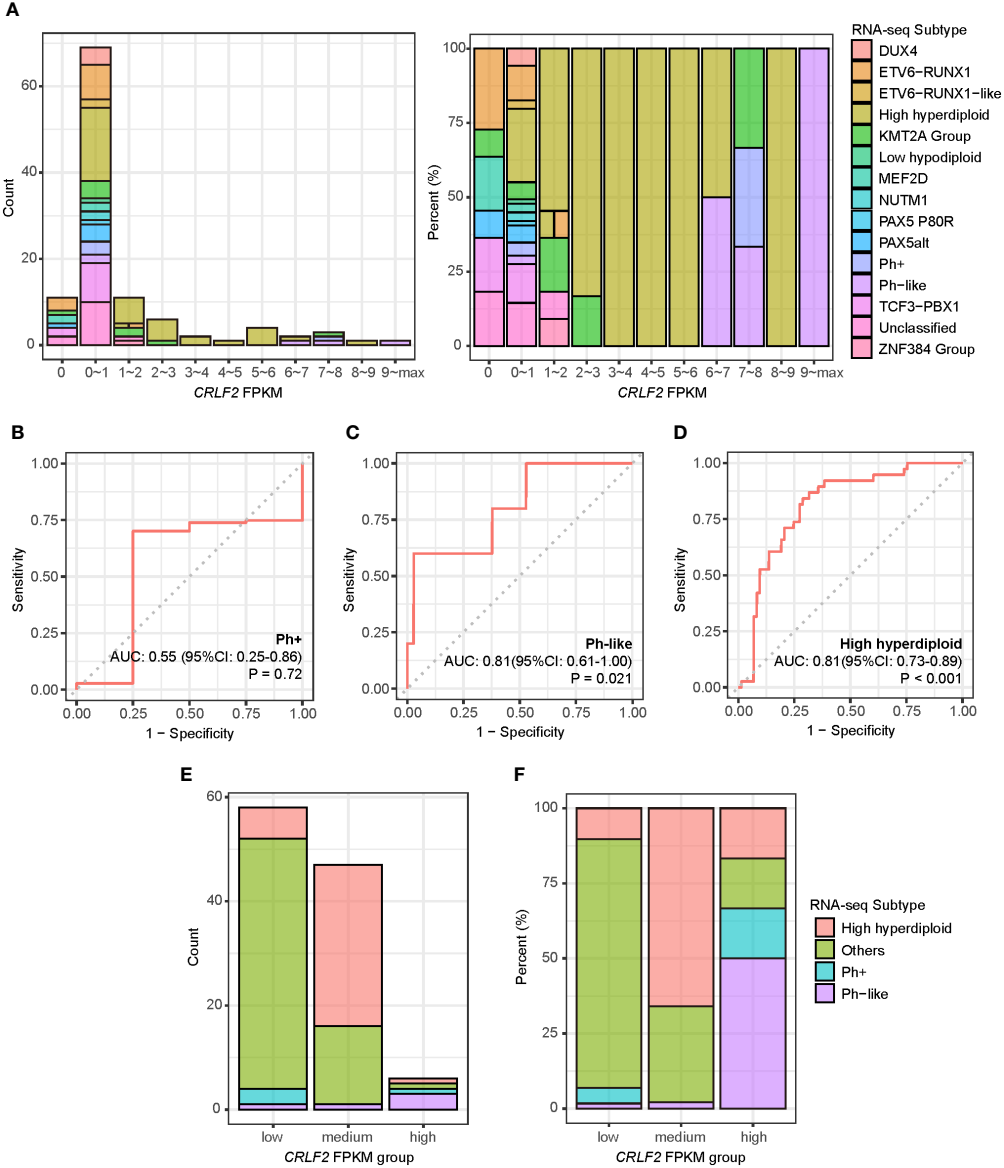
*CRLF2* expression and B-ALL subtypes of pediatric B-ALL patients in the Zhujiang Hospital cohort. **(A)** *CRLF2* expression in 111 B-ALL patients with RNA-seq B-ALL subtypes. **(B–D)** ROC curves for the correlation of *CRLF2* expression with the Ph+, Ph-like and high hyperdiploid subtypes. **(E, F)** RNA-seq subtypes in *CRLF2* FPKM groups. The Wilcoxon rank-sum test was applied to assess differences between 2 groups. The cutoff point between high/medium levels and medium/low levels were determined using the Youden index for *CRLF2* expression with the Ph-like and high hyperdiploid subtypes.

All the 111 treatment-naïve B-ALL patients in the Zhujiang Hospital cohort underwent RNA-seq using pre-treatment bone marrow samples, and fragments per kilobase of transcript per million (FPKM) were calculated through bioinformatics analysis. The expression level of the *CRLF2* gene was mainly within the interval of FPKM<1, i.e., generally low in the bone marrow samples of the treatment-naïve B-ALL patients. Some patients also manifested very high *CRLF2* expression, with a maximum FPKM of 75.1 (**Figure 1A**, left). Patients with high *CRLF2* expression mainly had the following B-ALL subtypes: KMT2A group, Ph+, Ph-like and high hyperdiploid (**Figure 1A**, right).

Subsequently, the *CRLF2* expression level was used to analyze the differences among the Ph+, Ph-like and high hyperdiploid subtypes. The expression level of the *CRLF2* gene was not the same between the Ph+ and Ph-like subtypes despite similar expression profiles. In addition, the expression level of the *CRLF2* gene in patients with the Ph-like and high hyperdiploid subtypes was significantly higher than that in other patients [**Figures 1C, D**, Ph-like: area under curve (AUC)=0.81, 95% confidence interval (CI): 0.611-1.000, P=0.021, high hyperdiploid: AUC=0.81, 95% CI: 0.728-0.894, *p*<0.001]. No correlation was found between the Ph+ subtype and *CRLF2* expression level (**Figure 1B**, *p*=0.72). The Youden indexes of *CRLF2* expression in predicting the Ph-like and high hyperdiploid subtypes were subsequently calculated (**Figure 1E**) (30), and the corresponding FPKM quantiles of *CRLF2* expression were set as the cutoffs (Ph-like cutoff point=94%, high hyperdiploid cutoff point=52%). *CRLF2* was assigned as a three-classification variable, namely *CRLF2*-high, *CRLF2*-medium and *CRLF2*-low (**Figure 1E**). Patients with *CRLF2*-high, *CRLF2*-medium and *CRLF2*-low accounted for 6% (Ph-like, n=3, high hyperdiploid, n=1, Ph+, n=1, KMT2A group, n=1), 42% (high hyperdiploid, n=31, Ph-like, n=1, other subtypes, n=15) and 52% (other subtypes, n=48, high hyperdiploid, n=6, Ph+, n=3, Ph-like, n=1), respectively, of all the patients (**Figure 1F**).

### Characteristics at diagnosis and minimal residual disease status during induction therapies among patients with different *CRLF2* expression levels

The clinical characteristics of the 3 groups of patients were analyzed. There were no significant differences in the incidence rates of concomitant testicular infiltration, central nervous system leukemia, intracranial hemorrhage, hepatosplenomegaly, and bone pain at the diagnosis of the disease in patients with different *CRLF2* expression levels (**Supplementary Table 1**).

**Figure 2:**
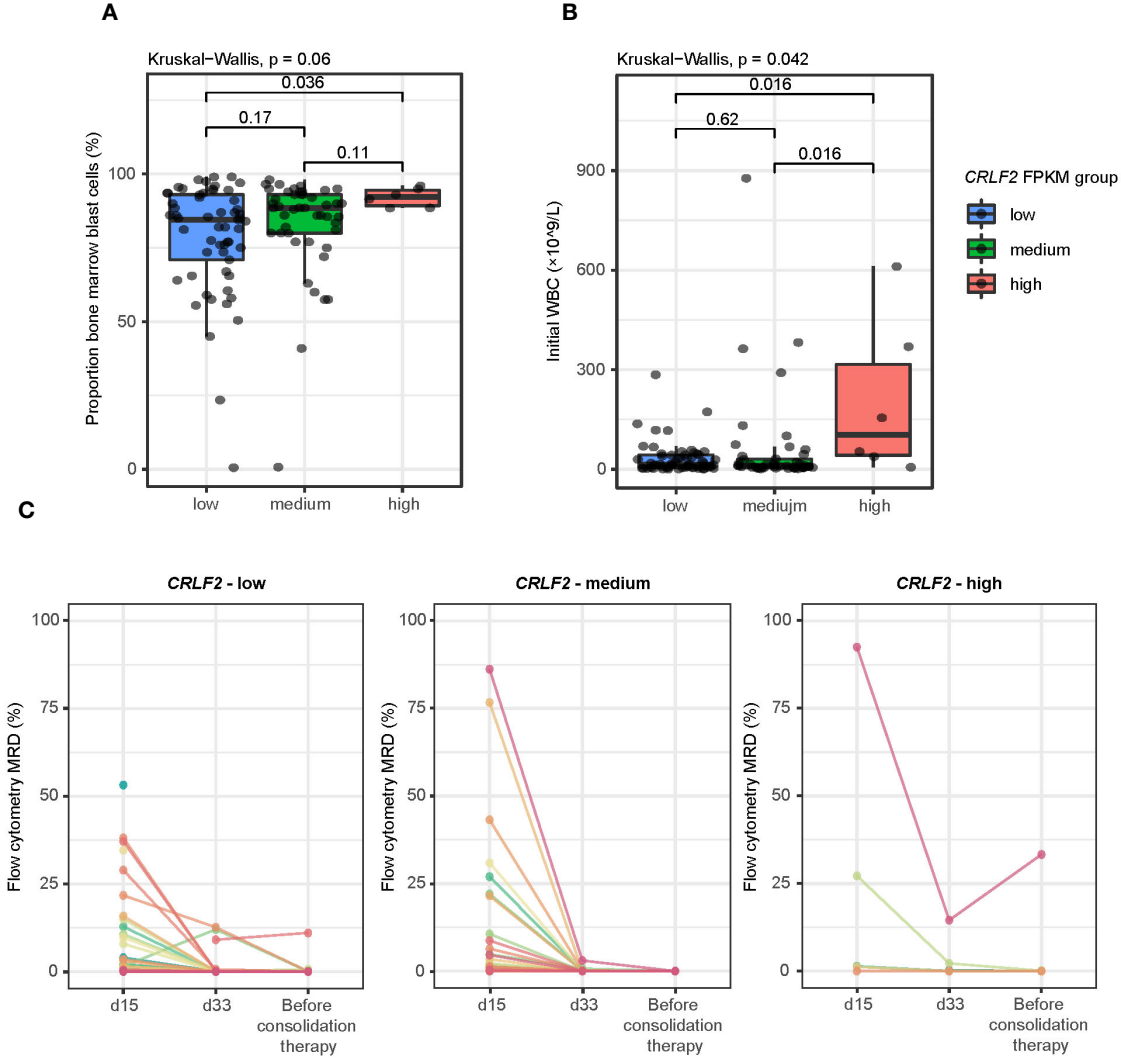
Association of *CRLF2* expression with blasts in BM, WBC, and flow cytometry MRD. **(A)** Proportion of bone marrow blast cells for patients with different *CRLF2* expression levels. **(B)** Initial WBC of patients with different *CRLF2* expression levels. **(C)** Flow cytometry MRD for different *CRLF2* expression levels. The Wilcoxon rank-sum test and Kruskal-Wallis test were applied to assess differences between 2 groups and more than 2 groups.

The proportion of bone marrow blast cells was not significantly different among the 3 groups of patients at diagnosis but was slightly higher in patients with *CRLF2*-high than in those with *CRLF2*-low (*CRLF2*-high *vs. CRLF2*-low, *p*=0.036, Kruskal-Wallis, *p*=0.06, **Figure 2A**). Similarly, the white blood cell count at initial visit was significantly higher in patients with *CRLF2*-high than in patients with *CRLF2*-low and *CRLF2*-medium (*CRLF2*-high *vs. CRLF2*-low, *p*=0.016, *CRLF2*-high *vs. CRLF2*-medium, *p*=0.016, Kruskal-Wallis, *p*= 0.042, **Figure 2B**).

The CR rate on 33^rd^ day was significantly lower in patients with *CRLF2*-high (33%) compared to those with *CRLF2*-medium (80%, *p* = 0.03) and *CRLF2*-low (76%, *p* = 0.046, global *p* = 0.062, **Figure S2**). Furthermore, the MRD status of the patients at different treatment time points was dynamically monitored by means of flow cytometry. The time points for monitoring were set as the 15^th^ day and 33^rd^ day after the start of induction therapies as well as before the start of consolidation therapy. The proportion of tumor cells in flow cytometry MRD decreased rapidly during induction therapy in patients with *CRLF2*-medium, and MRD remained negative by the time of assessment before consolidation therapy (a total of 43 patients available for assessment via flow cytometry MRD<0.1%). However, the patients with *CRLF2*-high and *CRLF2*-low had varying degrees of residual MRD, and the proportion of positive patients before consolidation therapy was 33.3% (n=2) and 5.5% (n=3), respectively (**Figure 2C**).

### Prognostic impact of *CRLF2*-high in the TARGET cohort

To further validate the association between the *CRLF2* expression level and poor prognosis, the publicly available TARGET cohort data were used as an externally independent validation cohort. The stratified cutoffs (quantiles) for the *CRLF2* expression level obtained from the Zhujiang Hospital cohort were employed to stratify the FPKM data of *CRLF2* in the TARGET cohort, followed by a survival analysis.

**Figure 3:**
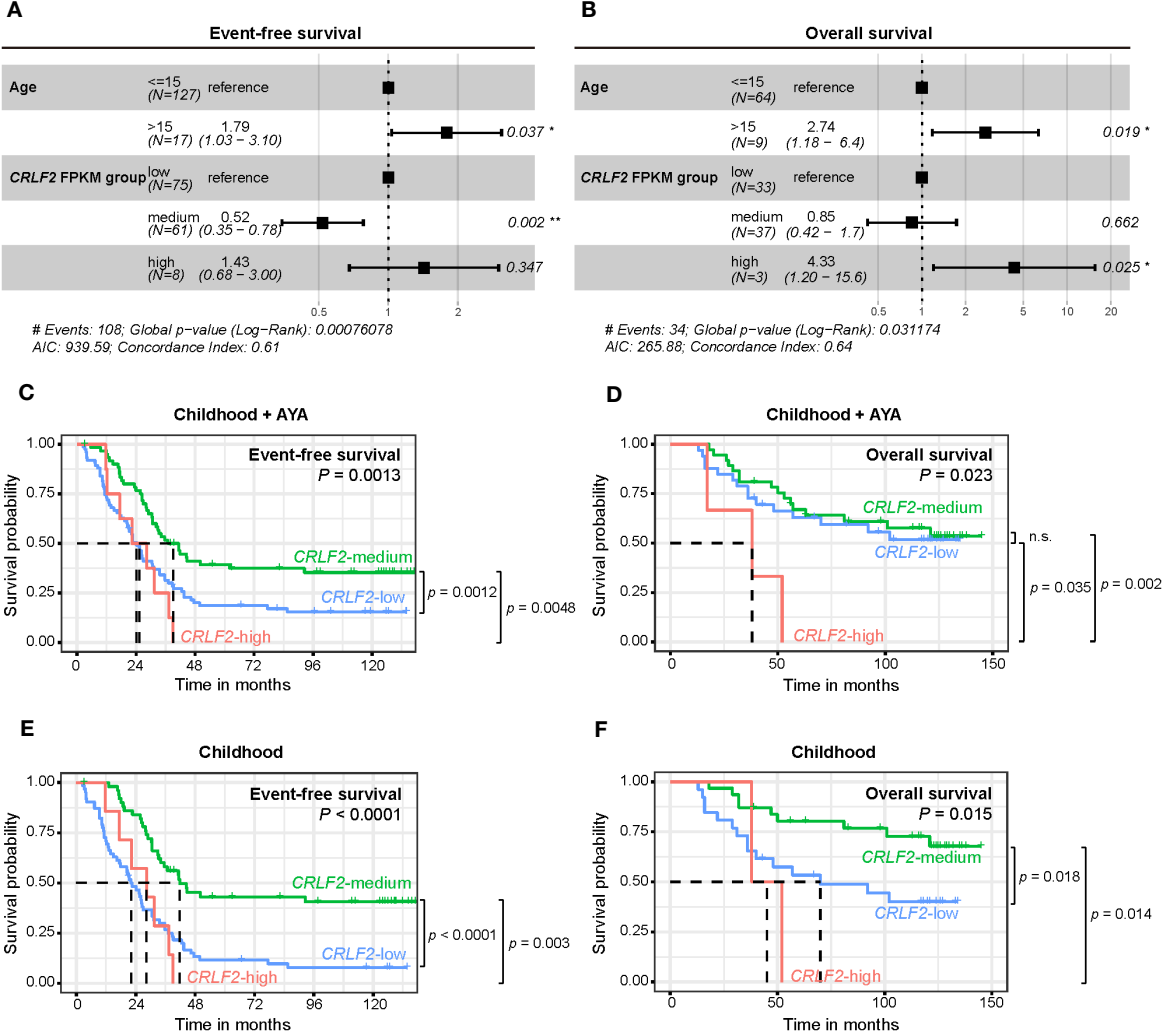
Survival analysis of pediatric B-ALL patients with *CRLF* expression in the TARGET cohort. **(A, B)** Effect of *CRLF2* expression levels on event-free survival (EFS, **A**) and overall survival (OS, **B**) in the TARGET cohort. Estimates are based on multivariate Cox regression. **(C, D)** Kaplan-Meier plots of OS and EFS for patients with different *CRLF2* expression levels in the TARGET cohort. **(E, F)** Kaplan-Meier plots of OS and EFS for pediatric patients with different *CRLF2* expression levels in the TARGET cohort.

Because only pediatric patients aged ≤15 years were recruited for the Zhujiang Hospital cohort and the age range of patients in the TARGET cohort was 2-30 years, age was included as a stratification factor in the multivariate Cox regression analysis (**Figures 3A, B**). Both age and *CRLF2* FPKM expression stratification served as independent prognostic factors for both event-free survival (EFS) and overall survival (OS) (EFS: C-index =0.61, *p*<0.001, OS: C-index =0.64, *p*=0.03). Age>15 years was a risk factor for both EFS and OS [EFS: hazard ratio (HR) =1.79, 95% CI: 1.03-3.10, *p*=0.037, OS: HR=2.74, 95% CI: 1.18-6.4, *p*=0.019]. However, *CRLF2* expression stratification of *CRLF2*-medium was not a risk factor for EFS (HR=0.52, 95% CI: 0.35-0.78, *p*=0.002), and *CRLF2*-high was a distinct risk factor for OS (HR=4.33, 95% CI: 1.20-15.6, *p*=0.025).

The effects of the *CRLF2* expression level on EFS and OS were further validated when plotting K-M curves. Without considering the influence of age on prognosis, patients with *CRLF2*-high and *CRLF2*-low had notably shorter EFS than those with *CRLF2*-medium (*CRLF2*-high *vs. CRLF2*-medium, *p*=0.0048, *CRLF2*-low *vs. CRLF2*-medium, *p*=0.0012, global *p*=0.0013, **Figure 3C**). The patients with *CRLF2*-high manifested an overtly shorter OS than did those with *CRLF2*-low and *CRLF2*-medium (*CRLF2*-high *vs. CRLF2*-low, *p*=0.035; *CRLF2*-high *vs. CRLF2*-medium, *p*=0.002, global *p*=0.023, **Figure 3D**); there was no significant difference in OS between the patients with *CRLF2*-medium and *CRLF2*-low.

Then, patients in the TARGET B-ALL cohort were selected as a childhood group (age ≤ 15 years old). The age range for the children in the childhood group from the TARGET B-ALL cohort was consistent with that for the children in the Zhujiang Hospital cohort. In the childhood group, both EFS and OS were markedly superior for patients with *CRLF2*-medium, compared with those for patients with *CRLF2*-low and *CRLF2*-high (EFS: *CRLF2*-medium *vs. CRLF2*-high, *p*=0.003, *CRLF2*-medium *vs. CRLF2*-low, *p*<0.0001, global *p*<0.0001, **Figure 3E**, OS: *CRLF2*-medium *vs. CRLF2*-high, *p*=0.014, *CRLF2*-medium *vs. CRLF2*-low, *p*=0.018, global *p*=0.015, **Figure 3F**). The results are consistent with the MRD status observed in the Zhujiang Hospital cohort.

### *CRLF2* expression levels among pediatric patients with *P2RY8-CRLF2* fusion

**Figure 4:**
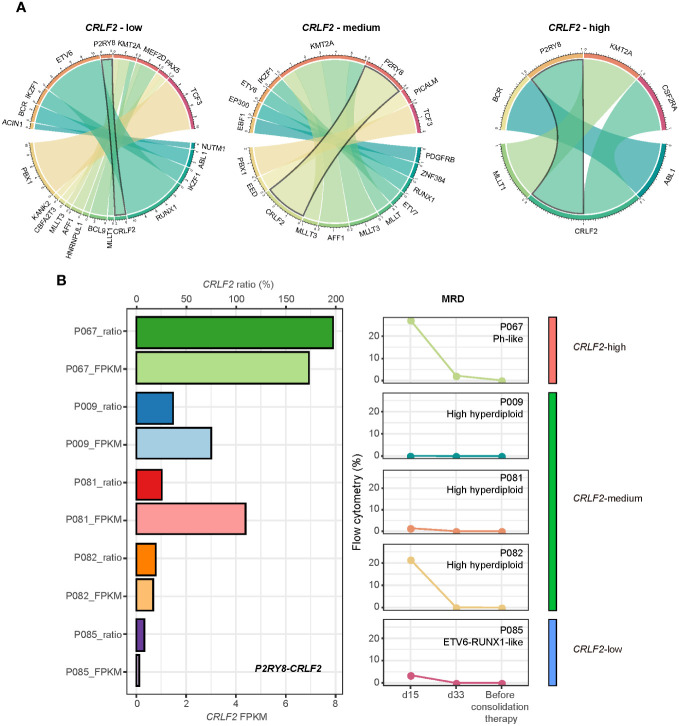
Association of *CRLF2* expression and *P2RY8-CRLF2* fusion. **(A)** Circos plot shows fusions in each *CRLF2* expression level. **(B)** RNA-seq (FPKM) and RQ-PCR (ratio) of *CRLF2* expression and dynamic changes in flow cytometry MRD for each *P2RY8-CRLF2* positive patient (n = 5).

In our cohort, fusion genes were also detected in 58 out of the 111 patients (53.3%). The fusion genes detected in patients with varying *CRLF2* gene expression levels are shown in **Figure 4A**. Although patients carried a wide range of fusion genes, *CRLF2*-related fusions were more prevalent in patients with *CRLF2*-high (2/6, 33.3%). In comparison, *P2RY8-CRLF2* fusion was the predominant *CRLF2*-related fusion (6/7, 85.7%). Among the patients carrying the *P2RY8-CRLF2* fusion, RQ-PCR was performed for 5 patients; the results are shown in **Figure 4B**. The FPKM of *CRLF2* was consistent with the RQ-PCR results (*CRLF2* ratio). The patient with the highest *CRLF2* expression level, namely, P067, had the Ph-like subtype, had a flow cytometry MRD of 0.1% as of consolidation therapy, and failed to achieve negative flow cytometry MRD. Patients P009, P081 and P082 with *CRLF2*-medium all had the high hyperdiploid subtype, and flow cytometry MRD was negative on the 33^rd^ day of induction therapy except for 1 patient who had an MRD of 0.1%; no residual tumor cells were detected in flow cytometry MRD in all 3 patients before consolidation therapy. Patient P085, with the lowest *CRLF2* expression level, had the ETV6-RUNX1-like subtype and had negative flow cytometry MRD results on the 33^rd^ day of induction therapy and thereafter. These results suggest that not all patients carrying the *P2RY8-CRLF2* fusion were *CRLF2*-high. Additionally, patients with the high hyperdiploid subtype and *P2RY8-CRLF2* fusion showed a better prognosis, as determined through flow cytometry MRD.

### Determination of Youden index for quantitative cutoffs of *CRLF2* gene expression by RQ-PCR

**Figure 5:**
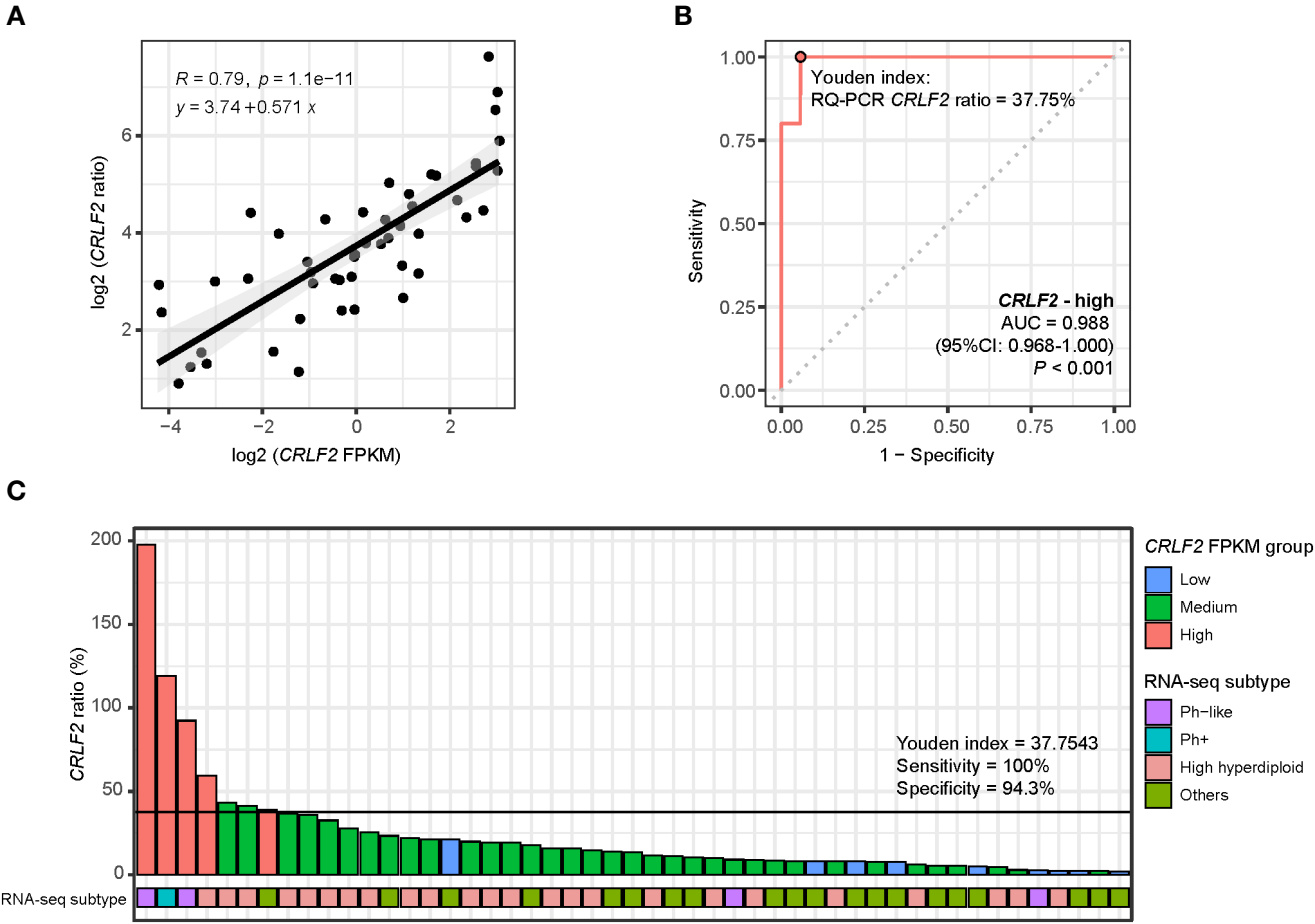
RQ-PCR for detection of *CRLF2* expression. **(A)** Scatter diagram and regression line for *CRLF2* expression measured by RQ-PCR (CRLF2_ratio) and RNA-seq (FPKM) (both in log 2 transformation). **(B)** ROC curves for the correlation of the RQ-PCR *CRLF2* ratio with *CRLF2*-high (in RNA-seq). **(C)** RQ-PCR *CRLF2* ratio and RNA-seq subtype for each patient (with RQ-PCR data, n = 49). The Wilcoxon rank-sum test and Kruskal-Wallis test were applied to assess differences between 2 groups and more than 2 groups. The cutoff points between *CRLF2*-high/-medium and *CRLF2*-medium/-low were determined using the Youden index of the RQ-PCR *CRLF2* ratio with *CRLF2*-high and *CRLF2*-low (*CRLF2* FPKM groups).

A total of 49 patients in the Zhujiang Hospital cohort underwent *CRLF2* expression testing through RNA-seq and RQ-PCR, and relatively consistent RQ-PCR results for *CRLF2* (*CRLF2* ratio) were obtained (R=0.79, *p*<0.0001, **Figure 5A**). Moreover, the cutoff value for *CRLF2*-high in RQ-PCR was determined, and the point value corresponding to the Youden index was used as a cutoff point as well (AUC=0.988, 95% CI: 0.968-1.000, *p*<0.001, Youden index cutoff point =37.75%, sensitivity =100%, and specificity =97%, **Figure 5B**). Then, the *CRLF2* ratios were arranged in descending order and classified by virtue of the RQ-PCR cutoff point, and 7 patients were classified as *CRLF2*-high (RQ-PCR data). There were 2 patients with the Ph-like subtype, 1 with the Ph+ subtype, 3 with the high hyperdiploid subtype, and 1 with the KMT2A subtype (**Figure 5C**). The 3 patients with the high hyperdiploid subtype had poor prognostic factors. Specifically, 1 patient had prednisone-poor response (PPR) on the 8^th^ day of prednisone treatment, 1 had flow cytometry MRD results that were positive by the 15^th^ day, and 1 carried mutations in genes (*NRAS* p.G13D, *PTPN11* p.S502A) in the RTK-RAS pathway.

## Discussion

*CRLF2* overexpression is involved in the pathogenesis and progression of B-ALL through the JAK/STAT signaling pathway (1–4). However, it is still unknown whether the prognosis of B-ALL patients can be predicted based on *CRLF2* expression levels (5, 31, 32). The proportion of patients with *CRLF2* overexpression varies substantially in previously published studies, and the proportion of patients with *CRLF2* overexpression among unscreened patients ranges from a minimum of 4.7% to a maximum of 25% (2, 4–7), implying that *CRLF2* overexpression needs to be defined by a more suitable cutoff value.

B-ALL can be categorized into different subtypes on the basis of genomic features and driver gene mutations, and such classification is of vital significance in risk stratification, treatment guidance and prognosis prediction. In this study, ALLSorts, defined by Gu et al., and RNA-seq analysis were used to identify 18 molecular subtypes (5, 31–33).

The exploration and analysis of the *CRLF2* expression level and molecular types of B-ALL revealed that the molecular types were heterogeneous in patients with different *CRLF2* expression levels. Patients with moderate upregulation of *CRLF2* mostly had the high hyperdiploid subtype, while those with *CRLF2* overexpression mainly had the Ph-like subtype. A close correlation between the Ph-like subtype and *CRLF2* expression has been reported. The studies by Martínez-Anaya et al. and Harvey et al. reported that more than 50% of patients with *CRLF2* overexpression have the Ph-like subtype (34, 35), a result that is consistent with the findings of this study. The high hyperdiploidy-associated upregulation of *CRLF2* expression primarily manifested as *CRLF2*-medium in our cohort, where more than half of this subgroup had the high hyperdiploid subtype. Such upregulation may be associated with the amplification of the X/Y chromosome where the *CRLF2* gene is located (**Supplementary Figures 1B, C**) (31, 36).

The majority of relevant studies have argued that *CRLF2* overexpression is correlated with the poor prognosis of B-ALL patients, especially those with high-risk B-ALL (2, 3, 31, 34, 37). However, some studies have argued that *CRLF2* overexpression has no impact on patient prognosis (5, 36). According to the baseline analysis of this cohort, the patients with *CRLF2*-high had a higher proportion of bone marrow blast cells and initial white blood cell counts. A high initial white blood cell count in childhood ALL is a recognized high-risk factor (38). Additionally, flow cytometry MRD decreased slowly or persistently positive during induction therapy in patients with *CRLF2*-high, indicating poor treatment response for such patients. The survival outcome for patients in the Zhujiang hospital cohort could not be analyzed due to the limited follow-up time. The publicly available childhood B-ALL data from the TARGET database were used to further validate the relationship between the *CRLF2* expression level and prognosis. The *CRLF2* expression level was defined in the TARGET cohort using the same methodology as that in the present cohort. Among B-ALL patients (pediatric + AYA), the patients with *CRLF2*-high had worse EFS and OS. Furthermore, pediatric patients with *CRLF2*-medium manifested a better prognosis, which is consistent with the better prognosis of pediatric patients with high hyperdiploid and X/Y chromosome amplification particularly (39–41). These results confirm the hypothesized relationship between *CRLF2* expression level and prognosis in this study: There is heterogeneity in patients with elevated *CRLF2* expression, that is, patients with *CRLF2*-high have a poor prognosis, and medium upregulation does not increase the prognostic risk.

*CRLF2* rearrangement is the most studied class of genetic alteration leading to *CRLF2* overexpression, mainly consisting of *P2RY8-CRLF2* and *IGH-CRLF2*. The former alteration results from local deletion on pseudoautosomes, an event that is related to Down’s syndrome-associated pediatric ALL (DS-ALL) (42). It was previously reported that *P2RY8-CRLF2* is mostly associated with a poor prognosis (2, 5, 37). Hence, the patients with *P2RY8-CRLF2* fusion in our cohort were also analyzed. In our cohort, *P2RY8-CRLF2* did not definitely lead to *CRLF2*-high. One patient with *P2RY8-CRLF2* fusion and *CRLF2*-high had the Ph-like subtype, and the flow cytometry MRD decreased slowly and persistently, suggesting a possible poor prognosis. However, most of the *P2RY8-CRLF2* patients with *CRLF2*-medium and *CRLF2*-low had the high hyperdiploid subtype and a better prognosis (3/4). It was speculated that *P2RY8-CRLF2* may be a minor subclone in such patients (43). *IGH-CRLF2* is another common type of *CRLF2* rearrangement. Russell et al. identified an immunoglobulin heavy chain gene (*IGH*) translocated to *CRLF2* at Xp22.3 or Yp11.3, forming the *IGH-CRLF2* fusion, in approximately 5% of BCP-ALL patients (44). In our cohort, *IGH-CRLF2* was found in only 1 (0.9%) patient. It has been reported that translocation patients (*IGH-CRLF2*) are older (median age: 16 years old), whereas deletion patients (*P2RY8-CRLF2*) are younger (median age: 4 years old) (2, 5, 37). In this study, the overall young age of our cohort (median age: 4 years old) may have led to fewer *IGH-CRLF2* patients.

In the exploration of cutoffs for the 3 categories of *CRLF2* expression, RNA-seq was adopted to detect the *CRLF2* expression level (FPKM) and molecular types obtained from transcriptional profiling analysis. However, RNA-seq, as a research-based assay, is not practical to carry out in the clinic. In contrast, RQ-PCR is a more accessible assay in clinical practice. For detecting *CRLF2* expression levels, RQ-PCR is faster, less expensive and more standardized than RNA-seq. The RNA-seq and RQ-PCR results were compared, and the detected *CRLF2* expression levels were highly consistent. Furthermore, defining *CRLF2*-high using RQ-PCR *CRLF2* ratio ≥37.75% as the cutoff yielded classification results that were consistent with those obtained using RNA-seq, thus laying a foundation for subsequent prospective studies on *CRLF2* overexpression for stratified treatment.

By analyzing the data from 111 Chinese pediatric B-ALL patients in our center, the classification of the *CRLF2* expression level into 3 categories more accurately described the prognosis and features of patients with various *CRLF2* expression levels. In the external validation cohort (TARGET cohort), patients with *CRLF2*-high had a significantly poor prognosis. The three-classification system for the *CRLF2* expression level detected by RQ-PCR in this study facilitates the rapid identification of patients with *CRLF2*-high in the clinic. For such patients, studies need to be conducted to further confirm and discover how to improve patient prognosis by intensifying chemotherapy or combining classic treatments with novel therapies.

## Data availability statement

The datasets presented in this study can be found in online repositories. The names of the repository/repositories and accession number(s) can be found in the article/**Supplementary Material**.

## Ethics statement

The studies involving humans were approved by the central ethics committee of Zhujiang Hospital, Southern Medical University. The studies were conducted in accordance with the local legislation and institutional requirements. Written informed consent for participation in this study was provided by the participants’ legal guardians/next of kin.

## Author contributions

DL: Data curation, Methodology, Resources, Writing – original draft. KY: Formal Analysis, Software, Visualization, Writing – original draft. LYu: Data curation, Resources, Writing – review & editing. LH: Data curation, Resources, Writing – review & editing. XL: Data curation, Resources, Writing – review & editing. LW: Data curation, Resources, Writing – review & editing. XX: Formal Analysis, Software, Writing – original draft. JZ: Investigation, Writing – original draft. QZ: Conceptualization, Methodology, Supervision, Writing – review & editing. LYa: Conceptualization, Methodology, Supervision, Writing – review & editing.

## Supplementary material

Supplementary Figure 1 Genomic alterations in pediatric B-ALL patients in the Zhujiang Hospital cohort. **(A)**. Gene mutation profiles for individual patients. **(B, C).** CRLF2-related structural variants **(B)** and X chromosome structural variation (**C**) with different CRLF2 expression levels.

Supplementary Figure 2 Survival and efficacy analysis in pediatric B-ALL patients in the Zhujiang Hospital cohort. CR rate in 33^rd^ with different *CRLF2* expression levels.

## References

[1] Cui L Gao C Wang CJ Zhao XX Li WJ Li ZG. Combined analysis of IKZF1 deletions and CRLF2 expression on prognostic impact in pediatric B-cell precursor acute lymphoblastic leukemia. Leuk Lymphoma (2021) 62(2):410–8. doi: 10.1080/10428194.2020.1832668 33054468

[2] Dou H Chen X Huang Y Su Y Lu L Yu J. Prognostic significance of P2RY8-CRLF2 and CRLF2 overexpression may vary across risk subgroups of childhood B-cell acute lymphoblastic leukemia. Genes Chromosomes Cancer (2017) 56(2):135–46. doi: 10.1002/gcc.22421 27637012

[3] Yano M Imamura T Asai D Moriya-Saito A Suenobu S Hasegawa D. An overall characterization of pediatric acute lymphoblastic leukemia with CRLF2 overexpression. Genes Chromosomes Cancer (2014) 53(10):815–23. doi: 10.1002/gcc.22190 24935070

[4] Ensor HM Schwab C Russell LJ Richards SM Morrison H Masic D. Demographic, clinical, and outcome features of children with acute lymphoblastic leukemia and CRLF2 deregulation: results from the MRC ALL97 clinical trial. Blood (2011) 117(7):2129–36. doi: 10.1182/blood-2010-07-297135 21106984

[5] Palmi C Vendramini E Silvestri D Longinotti G Frison D Cario G. Poor prognosis for P2RY8-CRLF2 fusion but not for CRLF2 over-expression in children with intermediate risk B-cell precursor acute lymphoblastic leukemia. Leukemia (2012) 26(10):2245–53. doi: 10.1038/leu.2012.101 22484421

[6] Yoda A Yoda Y Chiaretti S Bar-Natan M Mani K Rodig SJ. Functional screening identifies CRLF2 in precursor B-cell acute lymphoblastic leukemia. Proc Natl Acad Sci USA (2010) 107(1):252–7. doi: 10.1073/pnas.0911726107 PMC2806782 20018760

[7] Maciel ALT Barbosa TDC Blunck CB Wolch K MaChado AAL da Costa ES. IKZF1 deletions associate with CRLF2 overexpression leading to a poor prognosis in B-cell precursor acute lymphoblastic leukaemia. Transl Oncol (2022) 15(1):101291. doi: 10.1016/j.tranon.2021.101291 34826720 PMC8633010

[8] Gu M Jia Y Xu M Feng J Tian Z Ma X. Effects of different aberrations in the CRLF2 gene on the biological characteristics and drug sensitivities of Nalm6 cells. Int J Lab Hematol (2021) 43(3):441–9. doi: 10.1111/ijlh.13419 33615710

[9] Ge Z Gu Y Zhao G Li J Chen B Han Q. High CRLF2 expression associates with IKZF1 dysfunction in adult acute lymphoblastic leukemia without CRLF2 rearrangement. Oncotarget (2016) 7(31):49722–32. doi: 10.18632/oncotarget.10437 PMC5226542 27391346

[10] Shochat C Tal N Bandapalli OR Palmi C Ganmore I te Kronnie G. Gain-of-function mutations in interleukin-7 receptor-alpha (IL7R) in childhood acute lymphoblastic leukemias. J Exp Med (2011) 208(5):901–8. doi: 10.1084/jem.20110580 PMC3092356 21536738

[11] Pieters R De Lorenzo P Ancliffe P Aversa LA Brethon B Biondi A. Outcome of infants younger than 1 year with acute lymphoblastic leukemia treated with the interfant-06 protocol: results from an international phase III randomized study. J Clin Oncol (2019) 37(25):2246–56. doi: 10.1200/JCO.19.00261 31283407

[12] Li XY Li JQ Luo XQ Wu XD Sun X Xu HG. Reduced intensity of early intensification does not increase the risk of relapse in children with standard risk acute lymphoblastic leukemia - a multi-centric clinical study of GD-2008-ALL protocol. BMC Cancer (2021) 21(1):59. doi: 10.1186/s12885-020-07752-x 33435902 PMC7805214

[13] Chen S Zhou Y Chen Y Gu J. fastp: an ultra-fast all-in-one FASTQ preprocessor. Bioinformatics (2018) 34(17):i884–i90. doi: 10.1093/bioinformatics/bty560 PMC6129281 30423086

[14] Li H Durbin R. Fast and accurate short read alignment with Burrows-Wheeler transform. Bioinformatics (2009) 25(14):1754–60. doi: 10.1093/bioinformatics/btp324 PMC2705234 19451168

[15] Picard. Available at: http://broadinstitute.github.io/picard.

[16] McKenna A Hanna M Banks E Sivachenko A Cibulskis K Kernytsky A. The Genome Analysis Toolkit: a MapReduce framework for analyzing next-generation DNA sequencing data. Genome Res (2010) 20(9):1297–303. doi: 10.1101/gr.107524.110 PMC2928508 20644199

[17] Lai Z Markovets A Ahdesmaki M Chapman B Hofmann O McEwen R. VarDict: a novel and versatile variant caller for next-generation sequencing in cancer research. Nucleic Acids Res (2016) 44(11):e108. doi: 10.1093/nar/gkw227 27060149 PMC4914105

[18] Wang K Li M Hakonarson H. ANNOVAR: functional annotation of genetic variants from high-throughput sequencing data. Nucleic Acids Res (2010) 38(16):e164. doi: 10.1093/nar/gkq603 20601685 PMC2938201

[19] Thorvaldsdottir H Robinson JT Mesirov JP. Integrative Genomics Viewer (IGV): high-performance genomics data visualization and exploration. Brief Bioinform (2013) 14(2):178–92. doi: 10.1093/bib/bbs017 PMC3603213 22517427

[20] Kim D Paggi JM Park C Bennett C Salzberg SL. Graph-based genome alignment and genotyping with HISAT2 and HISAT-genotype. Nat Biotechnol (2019) 37(8):907–15. doi: 10.1038/s41587-019-0201-4 PMC7605509 31375807

[21] Dobin A Davis CA Schlesinger F Drenkow J Zaleski C Jha S. STAR: ultrafast universal RNA-seq aligner. Bioinformatics (2013) 29(1):15–21. doi: 10.1093/bioinformatics/bts635 23104886 PMC3530905

[22] Uhrig S Ellermann J Walther T Burkhardt P Frohlich M Hutter B. Accurate and efficient detection of gene fusions from RNA sequencing data. Genome Res (2021) 31(3):448–60. doi: 10.1101/gr.257246.119 PMC7919457 33441414

[23] Tian L Li Y Edmonson MN Zhou X Newman S McLeod C. CICERO: a versatile method for detecting complex and diverse driver fusions using cancer RNA sequencing data. Genome Biol (2020) 21(1):126. doi: 10.1186/s13059-020-02043-x 32466770 PMC7325161

[24] Anders S Pyl PT Huber W. HTSeq–a Python framework to work with high-throughput sequencing data. Bioinformatics (2015) 31(2):166–9. doi: 10.1093/bioinformatics/btu638 PMC4287950 25260700

[25] Schmidt B Brown LM Ryland GL Lonsdale A Kosasih HJ Ludlow LE. ALLSorts: an RNA-Seq subtype classifier for B-cell acute lymphoblastic leukemia. Blood Adv (2022) 6(14):4093–7. doi: 10.1182/bloodadvances.2021005894 PMC9327546 35482550

[26] Stanulla M Dagdan E Zaliova M Moricke A Palmi C Cazzaniga G. IKZF1(plus) defines a new minimal residual disease-dependent very-poor prognostic profile in pediatric B-cell precursor acute lymphoblastic leukemia. J Clin Oncol (2018) 36(12):1240–9. doi: 10.1200/JCO.2017.74.3617 29498923

[27] Gao J Liu WJ. Prognostic value of the response to prednisone for children with acute lymphoblastic leukemia: a meta-analysis. Eur Rev Med Pharmacol Sci (2018) 22(22):7858–66. doi: 10.26355/eurrev_201811_16411 30536331

[28] Xu LH Geng X Liao N Yang LH Mai HR Wan WQ. Prognostic significance of CNSL at diagnosis of childhood B-cell acute lymphoblastic leukemia: A report from the South China Children's Leukemia Group. Front Oncol (2022) 12:943761. doi: 10.3389/fonc.2022.943761 36033509 PMC9399517

[29] Brown PA Shah B Advani A Aoun P Boyer MW Burke PW. Acute lymphoblastic leukemia, version 2.2021, NCCN clinical practice guidelines in oncology. J Natl Compr Cancer Netw (2021) 19(9):1079–109. doi: 10.6004/jnccn.2021.0042 34551384

[30] Youden WJ. Index for rating diagnostic tests. Cancer (1950) 3(1):32–5. doi: 10.1002/1097-0142(1950)3:1<32::AID-CNCR2820030106>3.0.CO;2-3 15405679

[31] Chen IM Harvey RC Mullighan CG Gastier-Foster J Wharton W Kang H. Outcome modeling with CRLF2, IKZF1, JAK, and minimal residual disease in pediatric acute lymphoblastic leukemia: a Children's Oncology Group study. Blood (2012) 119(15):3512–22. doi: 10.1182/blood-2011-11-394221 PMC3325039 22368272

[32] Hassan NM Abdellateif MS Radwan EM Hameed SA Desouky EDE Kamel MM. Prognostic significance of CRLF2 overexpression and JAK2 mutation in Egyptian pediatric patients with B-precursor acute lymphoblastic leukemia. Clin Lymphoma Myeloma Leuk (2022) 22(6):e376–e85. doi: 10.1016/j.clml.2021.12.006 34987014

[33] Gu Z Churchman ML Roberts KG Moore I Zhou X Nakitandwe J. PAX5-driven subtypes of B-progenitor acute lymphoblastic leukemia. Nat Genet (2019) 51(2):296–307. doi: 10.1038/s41588-018-0315-5 30643249 PMC6525306

[34] Martinez-Anaya D Moreno-Lorenzana D Reyes-Leon A Juarez-Figueroa U Dean M Aguilar-Hernandez MM. Characterization of philadelphia-like pre-B acute lymphoblastic leukemia: experiences in mexican pediatric patients. Int J Mol Sci (2022) 23(17):9587. doi: 10.3390/ijms23179587 36076986 PMC9455471

[35] Harvey RC Mullighan CG Chen IM Wharton W Mikhail FM Carroll AJ. Rearrangement of CRLF2 is associated with mutation of JAK kinases, alteration of IKZF1, Hispanic/Latino ethnicity, and a poor outcome in pediatric B-progenitor acute lymphoblastic leukemia. Blood (2010) 115(26):5312–21. doi: 10.1182/blood-2009-09-245944 PMC2902132 20139093

[36] Rasekh EO Atef AM Khalil M Ebeid E Madney Y Hamdy N. Characterization of CRLF2 expression in pediatric B-cell precursor acute lymphoblastic leukemia. Clin Lab (2021) 67(1). doi: 10.7754/Clin.Lab.2020.200414 33491417

[37] Chiaretti S Brugnoletti F Messina M Paoloni F Fedullo AL Piciocchi A. CRLF2 overexpression identifies an unfavourable subgroup of adult B-cell precursor acute lymphoblastic leukemia lacking recurrent genetic abnormalities. Leukemia Res (2016) 41:36–42. doi: 10.1016/j.leukres.2015.11.018 26754556

[38] Schultz KR Pullen DJ Sather HN Shuster JJ Devidas M Borowitz MJ. Risk- and response-based classification of childhood B-precursor acute lymphoblastic leukemia: a combined analysis of prognostic markers from the Pediatric Oncology Group (POG) and Children's Cancer Group (CCG). Blood (2007) 109(3):926–35. doi: 10.1182/blood-2006-01-024729 PMC1785141 17003380

[39] Lee SHR Antillon-Klussmann F Pei D Yang W Roberts KG Li Z. Association of genetic ancestry with the molecular subtypes and prognosis of childhood acute lymphoblastic leukemia. JAMA Oncol (2022) 8(3):354–363. doi: 10.1001/jamaoncol.2021.6826 35084434 PMC8796058

[40] Li JF Dai YT Lilljebjorn H Shen SH Cui BW Bai L. Transcriptional landscape of B cell precursor acute lymphoblastic leukemia based on an international study of 1,223 cases. Proc Natl Acad Sci USA (2018) 115(50):E11711–E20. doi: 10.1073/pnas.1814397115 PMC6294900 30487223

[41] Paulsson K Lilljebjorn H Biloglav A Olsson L Rissler M Castor A. The genomic landscape of high hyperdiploid childhood acute lymphoblastic leukemia. Nat Genet (2015) 47(6):672–6. doi: 10.1038/ng.3301 25961940

[42] Mullighan CG Collins-Underwood JR Phillips LA Loudin MG Liu W Zhang J. Rearrangement of CRLF2 in B-progenitor- and Down syndrome-associated acute lymphoblastic leukemia. Nat Genet (2009) 41(11):1243–6. doi: 10.1038/ng.469 PMC2783810 19838194

[43] Morak M Attarbaschi A Fischer S Nassimbeni C Grausenburger R Bastelberger S. Small sizes and indolent evolutionary dynamics challenge the potential role of P2RY8-CRLF2-harboring clones as main relapse-driving force in childhood ALL. Blood (2012) 120(26):5134–42. doi: 10.1182/blood-2012-07-443218 PMC4194314 23091296

[44] Russell LJ Capasso M Vater I Akasaka T Bernard OA Calasanz MJ. Deregulated expression of cytokine receptor gene, CRLF2, is involved in lymphoid transformation in B-cell precursor acute lymphoblastic leukemia. Blood (2009) 114(13):2688–98. doi: 10.1182/blood-2009-03-208397 19641190

