# Supplementary figures and images for: Poor outcome of pediatric B-cell acute lymphoblastic leukemia associated with high level of CRLF2 gene expression in distinct molecular subtypes
Source: Front Oncol. 2023 Nov 7;13:1256054. doi: 10.3389/fonc.2023.1256054 (PMC10661883; doi:10.3389/fonc.2023.1256054)

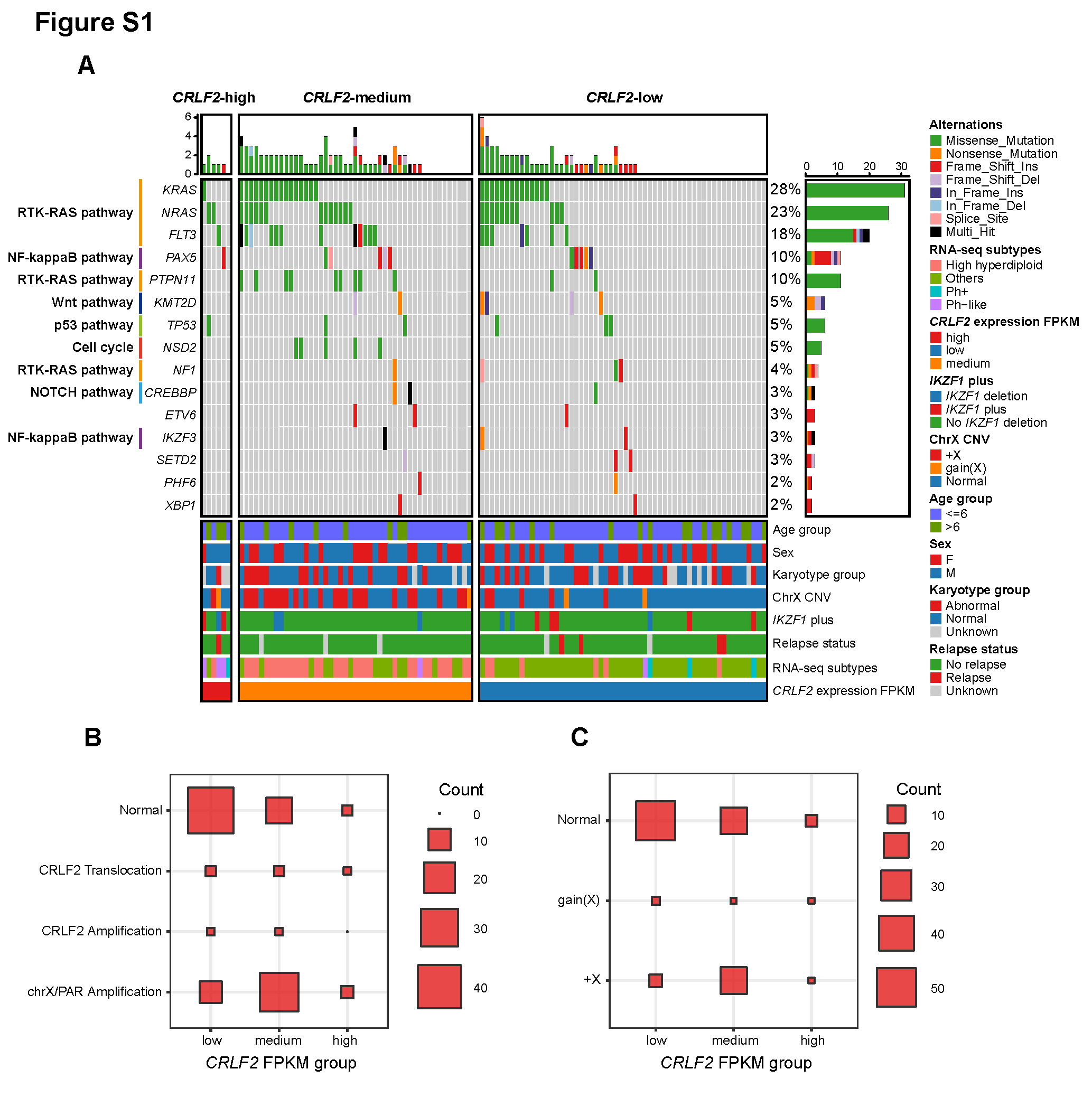

Supplement: Supplementary Figure 1 — Genomic alterations in pediatric B-ALL patients in the Zhujiang Hospital cohort. (A). Gene mutation profiles for individual patients. (B, C). CRLF2-related structural variants (B) and X chromosome structural variation (C) with different CRLF2 expression levels. [file Image_1.tif]

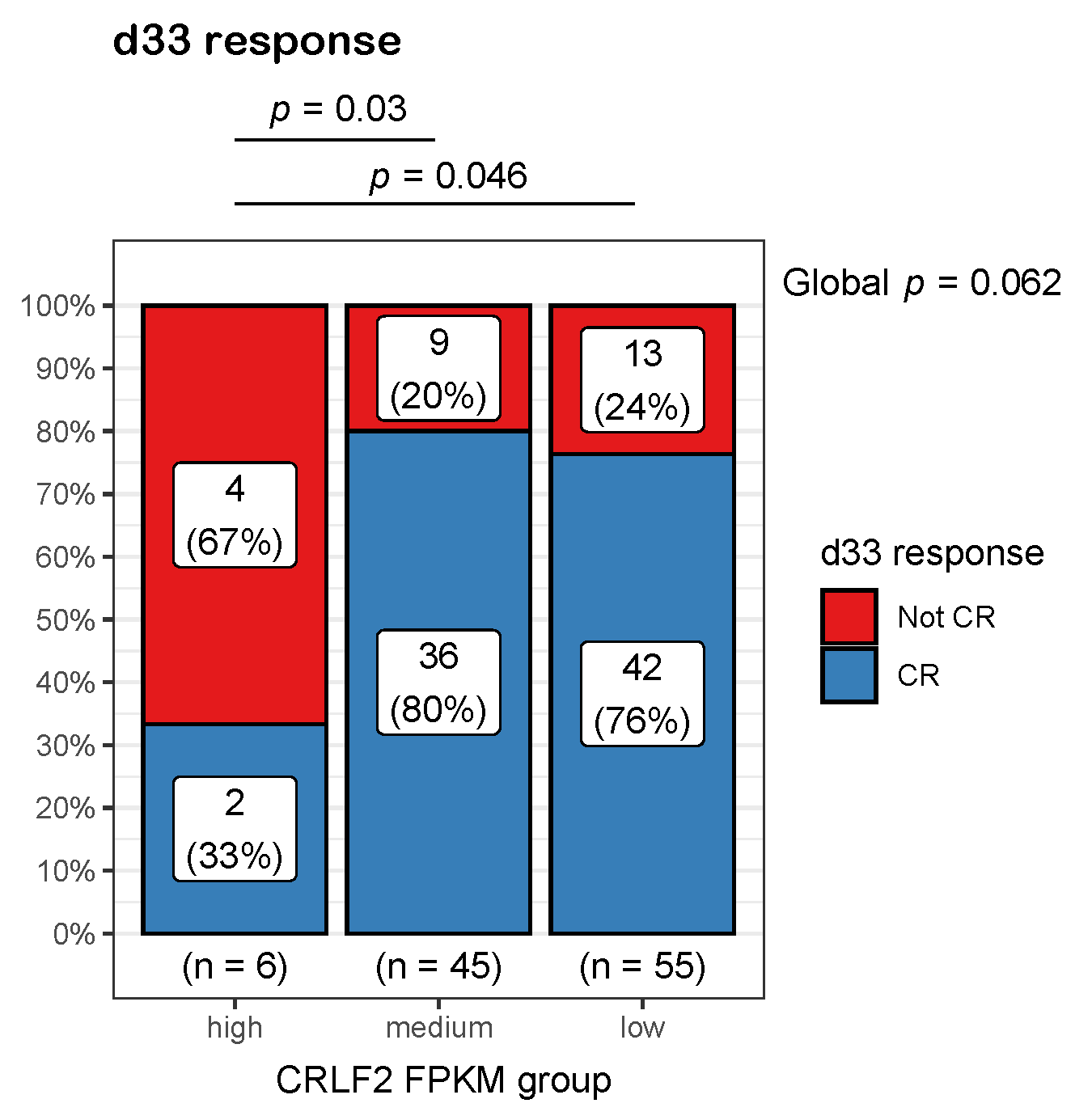

Supplement: Supplementary Figure 2 — Survival and efficacy analysis in pediatric B-ALL patients in the Zhujiang Hospital cohort. CR rate in 33rd with different CRLF2 expression levels. [file Image_2.tif]
