# Supplementary material for: Poor outcome of pediatric B-cell acute lymphoblastic leukemia associated with high level of CRLF2 gene expression in distinct molecular subtypes
Source: Front Oncol. 2023 Nov 7;13:1256054. doi: 10.3389/fonc.2023.1256054 (PMC10661883; doi:10.3389/fonc.2023.1256054)
Supplement: Supplementary file 3 [file Table_1.docx]

**Supplementary Table 1. Clinical baseline of patients with different *CRLF2* expression levels**

| **Characteristics** | **Total** | ***CRLF2* expression level** | | | **P-value** |
| --- | --- | --- | --- | --- | --- |
|  |  | *CRLF2*-  low  N (%) | *CRLF2*-  medium  N (%) | *CRLF2*-  high  N (%) |  |
| Central nervous system leukaemia | | | | | 0.081 |
| Positive | 3 | 2(3.4%) | 0(0.0%) | 1(16.7%) |  |
| Negative | 108 | 56(96.6%) | 47(100%) | 5(83.3%) |  |
| Testicular leukaemia | | | | | 1 |
| Positive | 1 | 1(1.7%) | 0(0.0%) | 0(0.0%) |  |
| Negative | 110 | 57(98.3%) | 47(100%) | 6(100%) |  |
| Intracranial haemorrhage | | | | | 0.478 |
| Positive | 1 | 0(0.0%) | 1(2.1%) | 0(0.0%) |  |
| Negative | 110 | 58(100%) | 46(97.9%) | 6(100%) |  |
| Hepatosplenomegaly | | | | | 0.781 |
| Positive | 58 | 30(48.3%) | 23(48.9%) | 4(66.7%) |  |
| Negative | 53 | 28(51.7%) | 24(51.1%) | 2(33.3%) |  |
| Bone pain | | | | | 0.564 |
| Positive | 25 | 11(19.0%) | 12(25.5%) | 2(33.3%) |  |
| Negative | 86 | 47(81.0%) | 35(74.5%) | 4(66.7%) |  |
