# Supplementary material for: Poor outcome of pediatric B-cell acute lymphoblastic leukemia associated with high level of CRLF2 gene expression in distinct molecular subtypes
Source: Front Oncol. 2023 Nov 7;13:1256054. doi: 10.3389/fonc.2023.1256054 (PMC10661883; doi:10.3389/fonc.2023.1256054)
Supplement: Supplementary file 4 [file Table_2.docx]

**Supplementary Table 2. Characteristics of the TARGET cohort.**

| **Characteristics** | | **Total (n = 144)** | |
| --- | --- | --- | --- |
| Sex (n, %) | Female | 71 | 49% |
|  | Male | 73 | 51% |
| Race (n, %) | Asian | 5 | 3% |
|  | Black or African American | 17 | 12% |
|  | Unknown | 23 | 16% |
|  | White | 99 | 69% |
| Ethnicity (n, %) | Hispanic or Latino | 33 | 23% |
|  | Not Hispanic or Latino | 99 | 69% |
|  | Unknown | 12 | 8% |
| Type (n, %) | B-Precursor | 132 | 92% |
|  | B-Cell ALL | 12 | 8% |
| Age (years) | median (Range) | 7 | (2-30) |
| WBC (×10^9^/L) | median (IQR) | 28.55 | (11.95-84.2) |
| CNS (n, %) | CNS 1 | 115 | 80% |
|  | CNS 2 | 25 | 17% |
|  | CNS 3 | 4 | 3% |
| RNA-seq subtype (n, %) | DUX4 | 2 | 1% |
|  | ETV6-RUNX1 | 15 | 10% |
|  | ETV6-RUNX1-like | 4 | 3% |
|  | High hyperdiploid | 23 | 16% |
|  | High Sig | 4 | 3% |
|  | NUTM1 | 2 | 1% |
|  | HLF | 3 | 2% |
|  | iAMP21 | 4 | 3% |
|  | KMT2A Group | 3 | 2% |
|  | Low hyperdiploid | 1 | 1% |
|  | MEF2D | 4 | 3% |
|  | PAX5alt | 15 | 10% |
|  | Ph | 5 | 3% |
|  | Ph-like | 28 | 19% |
|  | TCF3-PBX1 | 17 | 12% |
|  | Unclassified | 6 | 4% |
|  | ZNF384 Group | 13 | 9% |
| Protocol (n, %) | AALL0232 | 91 | 63% |
|  | AALL0331 | 52 | 36% |
|  | 9906 | 1 | 1% |
| *CRLF2* expression group (n, %) | low | 75 | 52% |
|  | medium | 61 | 42% |
|  | high | 8 | 6% |

*ALL* acute lymphoblastic leukaemia, *WBC* white blood cell, *CNS* central nervous system.
